# Mothers’ Experiences Raising Children With Complex Care Needs in Rural Settings: A Qualitative Study

**DOI:** 10.1177/30502225251332024

**Published:** 2025-05-02

**Authors:** Hélène Corriveau, Penelopia Iancu, Anik Dubé, Vickie Plourde

**Affiliations:** 1 Université de Moncton, NB, Canada

**Keywords:** children, complex care needs, mothers, community social pediatrics, rural settings

## Abstract

**Introduction:**

Having a child with complex care needs (CCNs) can significantly impact families’ daily life structure. High caregiving responsibilities perceived by mothers and limited access to services—especially in rural settings—can affect their mental health and lead to caregiver burnout.

**Objectives:**

This study aimed to explore the challenges faced by mothers of children with CCNs living in a rural setting. Methods: Eight mothers, recruited through a Community Social Pediatrics Centre in New Brunswick (CA), were individually interviewed between July 2020 and January 2021. Interviews were transcribed and analyzed using a thematic analysis method.

**Results:**

Results highlight challenges experienced by mothers in accessing care and formal resources for their children. Both the lack of support and challenges faced by the child increased mothers’ burden.

**Discussion:**

Future research and intervention are warranted to better identify and meet the needs of mothers having a child with CCNs and living in rural settings.

## Introduction

Having a child with complex care needs (CCNs)—defined as children with multidimensional care needs that include a combination of social, medical, and behavioral interventions^1,2^—can significantly impact parents’ quality of life and their family structure.^3^ Over the years, gender-based research has shown that mothers with children with CCNs report more responsibilities than fathers regarding their child’s care and development,^4^ leaving little to no time for personal activities or self-care opportunities.^5,6^ The high levels of responsibility and involvement shown by these mothers can negatively affect their mental health and lead to caregiver burnout.^7^ Quantitative studies have shown higher levels of distress, depression, anxiety, and suicidal thoughts among mothers of children living with a disability^8^ as well as a greater caregiver burden and a lower quality of life.^9^

Since the late 1980s, pediatric care has shifted toward a more integrated family-centered approach.^10,11^ Integrated pediatric services are associated with multiple positive outcomes, including increased accessibility to healthcare services and greater family involvement in decision-making.^12^ However, despite these promising results, children with CCNs and families residing in rural settings often face greater barriers to accessing these services compared to their urban counterparts. Due to geographic location and limited local resources, rural families must travel longer distances to access specialized care.^13,14^ Additional challenges, such as travel cost, weather conditions, and long wait times for appointments, further hinder access to care.^14,15^ These factors—combined with social loneliness and financial hardship^16^—can contribute to poorer health outcomes^17^ and a higher caregiving burden.

To address these disparities, a Community Social Pediatrics (CSP) center was established in 2017 in 3 rural communities (Memramcook, Bouctouche, and Richibucto) in South-East New Brunswick (Canada) adopting an integrated social medicine approach to pediatric care.^18,19^ CSP is a promising approach for families living in rural settings, where access to healthcare and social services can be limited.^15,20^ The clinical team includes mainly pediatricians and social workers, collaborating with other health professionals and legal consultants, to offer a more accessible and holistic model of care, and to help reduce the burden of family caregivers living in rural communities.^21^

The Informal Caregiving Integrative Model (ICIM)^22^ provides a framework for understanding the factors that contribute to the caregiver burden. According to this model, multiple factors have been linked to the caregiver’s burden, including the caregivers’ health determinants (eg, services, informal support, and sociocultural environment), the caregiving setting (eg, primary and secondary stressors) and their individual characteristics (eg, sociodemographic, psychological and physical state). While the literature on caregivers’ burden, specifically related to parents of children with CCNs, remains limited, existing evidence suggests that caregiver burnout is prevalent and is often pronounced for parents of a child with CCNs. A recent scoping review^23^ reported that several factors can contribute to caregivers’ burden, including the parents’ sociodemographic and psychological characteristics, physical health, coping strategies, as well as child-related factors such as the severity of the child’s condition, the specific diagnosis, and the time since diagnosis. Additionally, the environmental context, including the availability of social support and the quality of relationships, can play a critical role in shaping the caregiver burden.^23^

However, there is a notable gap in research focusing specifically on mothers caring for children with CCNs, particularly in rural context. The application of the ICIM in these contexts remains unclear, especially given that rural caregivers may face unique challenges such as geographic location, reliable transportation, and specialist availability.^15^ Furthermore, few studies have used a qualitative approach^24^ to explore mothers’ caregiving experiences. It is essential to understand the mother’s experience, as it will allow healthcare providers to enhance access to care and tailor community health services to better meet their needs. The aim of this qualitative study was to focus on mothers caring for a child with CCNs in a rural setting and to explore their perspectives on (1) the social and health-related challenges faced by their child and (2) their own challenges and burden.

## Methods

### Study Design

A case study approach was adopted to explain, describe, and explore mothers’ experiences.^25^ This current study is part of a larger-scale research project on the Community Social Pediatrics (CSP) Centre in rural communities located in Memramcook (5028 inhabitants^26^), Bouctouche (2513 inhabitants^26^), Richibucto (1464 inhabitants^26^). This research project has received ethical approval from 2 research ethics committees (Vitalité health authority and Université de Moncton ethics board). Data presented will focus on qualitative semi-structured interviews conducted during COVID-19 (July 2020 and January 2021) with mothers who received services from the CSP Centre.

### Participants

Families receiving services from the CSP Centre were invited by the CSP clinical staff to take part in a larger-scale research project. Upon consent, families’ contact information (phone number and email address) was shared with the research team. During the recruitment period, 72 families were receiving services from the CSP center. The CSP clinical staff sent the contact information of a total of 25 families who expressed interest in participating in the research project. A research team member (HC, female, MSW, research associate, with extended experience in qualitative research) contacted each family and asked them if they were willing to participate in the quantitative component of the larger-scale study. Fourteen families (out of 25; participation rate = 56%) agreed to complete the questionnaires for the quantitative part of the study. A few months after completion, those 14 families were contacted by telephone by a research team member (HC) and invited to take part in the second component of the study, an individual semi-structured interview. In total, 8 mothers (out of 14; participation rate = 57%) agreed to participate (1 parent refused, and the research team member was unable to reach the 5 other parents).

### Measures

#### Sociodemographic Information

We developed and administered—prior to the semi-structured interview—a sociodemographic questionnaire to participants to document their age, highest level of education, family structure, and family income. Additional questions regarding their children, such as the date services started, reasons for services, gender, and medication, were also asked to participants.

#### Semi-Structured Interviews

The individual interviews (N = 8) were conducted during COVID-19, and due to sanitary restrictions, they could only be conducted over the phone. Participants selected the language for the interviews: 7 chose French, and 1 chose English. Prior to each interview, the research team member (HC) obtained oral informed consent from each participant to take part in this study. The interview guide was developed by the research team members for the purpose of the research project and comprised 5 sections: changes observed in their children, living conditions of the family, children’s rights, engagement in children’s education, and satisfaction with services received through CSP (Interview guide is provided in a Supplemental File). Interviews lasted between 30 and 60 minutes and were audio recorded. Each participant received a gift card ($25) to compensate for their time.

### Statistical Analysis

#### Sociodemographic Profile

SPSS (version 29.0.0.0) was used to conduct analyses. Descriptive analyses were conducted for participants’ age, highest level of education, family structure, and family income, and for children’s age, gender, reasons for services, medication, and amount of time they had been receiving services.

#### Qualitative Analysis

Interviews were transcribed in full and then analyzed. Six research team members carried out an initial analysis of all the interviews to determine the main themes, using a deductive approach based on the interview guide.^27^ After the identification of the main themes, 2 subgroups of 3 members were formed within the team, and a first coding was performed independently by each member of the group using a thematic analysis approach to identify subthemes based on participants’ verbatim.^28^ Full agreement was ensured within subgroups. Lastly, the team met to validate and proceed to the triangulation of coding for all 8 interviews. Since most data were in French, a back translation process^29^ was used for the verbatim extracts to English. To ensure confidentiality and anonymity, fictitious names were assigned to each participant.

## Results

### Family Circumstances

**Figure 1.**
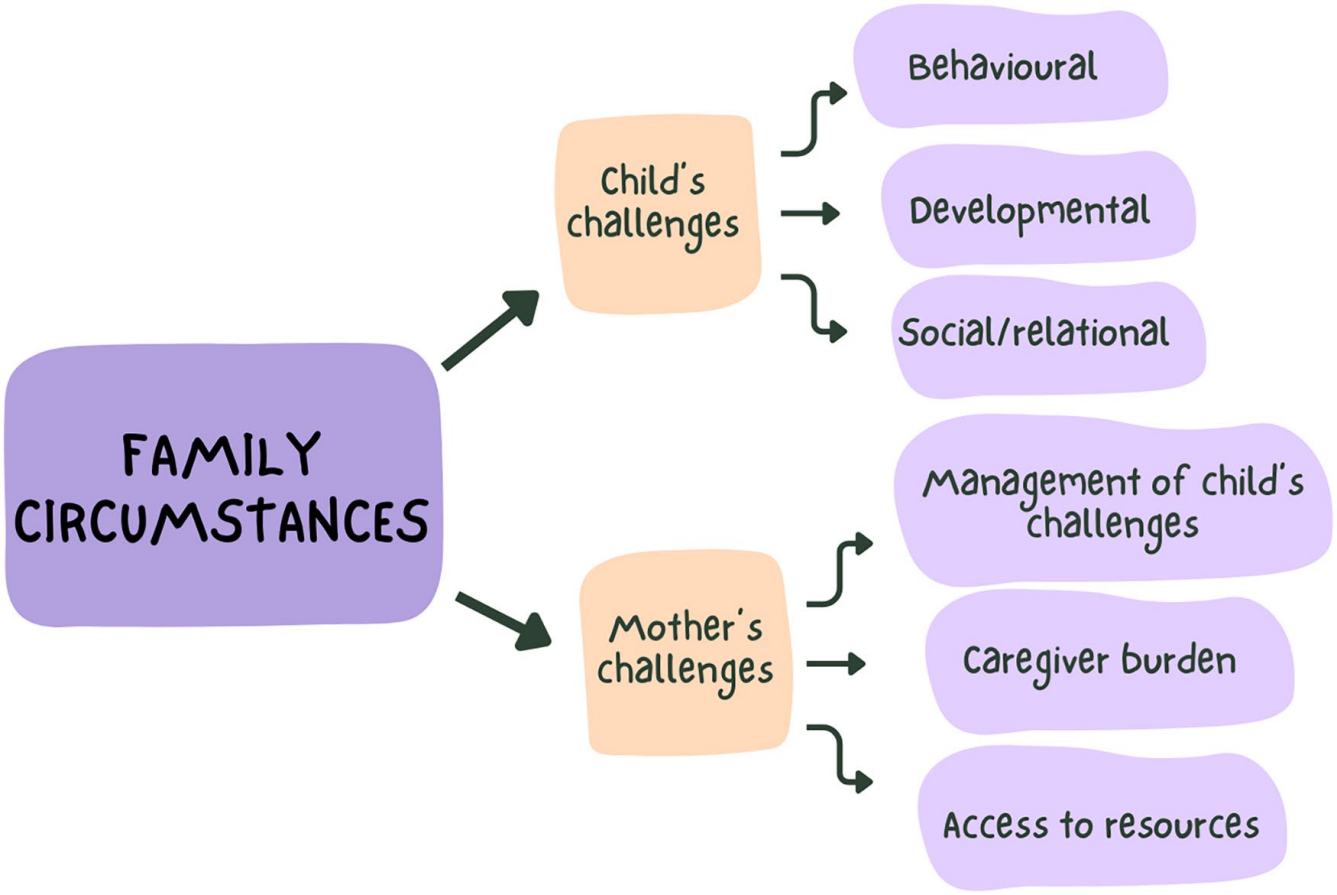
Coding tree- family circumstances.

Thematic analyses highlighted 2 main themes: (1) family circumstances (child’s challenges and mothers’ challenges), and (2) CSP services received. Results presented in this article will focus on the first theme. Figure 1 illustrates the coding tree.

### Participant Profile

For 6 mothers, 2 of their children were receiving services from the CSP Centre, while 2 others were receiving services for only 1 child. On average, children had been receiving services for 1.91 years (SD = 0.65). Families received clinical services primarily for a medical diagnosis (physical or mental) and/or because of toxic stress in the family home (eg, tense family atmosphere, poor parenting skills) impacting their children’s well-being. Children were either living with both parents (71.4%) or in a single-parent family (28.6%). The average age for mothers participating in the study was 41.79 years old (SD = 5.31), 50% had a college diploma, 38% had a university degree, and 12% had a high school diploma. Three mothers were unemployed, and 5 mothers reported having a family revenue of $60 000 or more in the past year. The children’s age was on average 10.12 years (SD = 4.37), with 57.1% boys and 42.9% girls. In the past 12 months, 7 children had taken medication to treat a mental health problem, 1 had medication to treat a physical health problem, and 1 had taken medication to treat both mental and physical health problems.

### Child’s Challenges

Three areas for children’s challenges were identified as subthemes: (1) behavioral, (2) developmental, and (3) social/relational. Six mothers identified at least 1 challenge in the behavioral category. For 5 mothers, behavioral challenges were reported as hyperactivity, attention/sensory management, impulsivity, or aggressivity. For instance, Anna explained that her 7-year-old son had a hard time staying seated during mealtime. Another participant, Charlotte, reported that her 7-year-old son presents sensitivity to sound at school, which makes him easily distracted. Finally, Emma expressed that her son’s impulsivity made it difficult for him to be aware of dangerous situations and led her to reconsider leaving him home alone even though he is 14 years old. For 2 other participants, their children were showing aggressive behaviors (eg, verbal aggression).

Developmental challenges, reported by 4 mothers, included lack of autonomy, food rigidities, and speech delays. Three mothers found their child had difficulties being independent or being able to perform age-appropriate tasks. For example, Emma expressed how worried she was when her 14-year-old son tried to cook on his own, because he was unaware of the danger. As for food rigidities, 3 participants mentioned their child had a hard time trying or integrating new foods into their diet. Anna also discussed her son’s speech delays and the difficulties and frustrations encountered when he tries to express himself.

Finally, 6 mothers reported that their child had social/relational challenges. For instance, Sophie sought services for her 11-year-old daughter, who was showing difficulties with anxiety management. She was worried that her difficulty in managing her emotions could have a negative and long-term impact on her daughter’s friendships. For the other 2 participants, Anna and Charlotte, their child is showing difficulty with social skills (ie, social cues, sharing toys), which impacted their ability to establish relationships with pairs. Finally, Valery’s son, who is a teenager, doesn’t want his friends to know he needs accommodations in classes (ie, personalized learning plans) because he is worried that it could affect his relationships with them.

### Mothers’ Challenges

All mothers who participated in the study reported at least one of the following challenges (3 subthemes): (1) management of children’s challenges, (2) caregiver burden, and (3) access to resources. Seven mothers said they had a hard time managing, understanding, and accepting their child’s challenges. For example, Anna explains:

He hates when I put the timer, it upsets him at the highest level, it insults him, but I don’t have a choice, I put it on because otherwise the meal would last 2 hours and he’d still be at the table, so that, it’s still difficult to deal with.

For Sophie, managing her daughter’s eating habits (ie, always reaching for snacks) was difficult because she didn’t want to aggravate the situation: “I had a hard time dealing with that because I didn’t want to give her a body image issue but at the same time, I wanted her to be aware of the choices she was making.”

For some participants, the management of their child’s challenges was complexified by their own difficulty in understanding their child’s new diagnosis: “Well I found it difficult when Mathieu was diagnosed with ADHD, it was an adjustment for me to understand the behavior (. . .) to try to help him through that” (Laura). For others, managing the child’s challenges was complexified by divergent views on parenting and inconsistent use of practices between parents. One mother stated that she sometimes felt like her husband was not following through with the psychologist’s recommendations, which led to issues in managing their child’s challenges. Finally, one participant found it harder to accept her son’s way of dealing with his emotions, since he: “wanted to withdraw and be alone, that for me was a challenge” (Valery).

Six mothers highlighted not only the intense caregiver burden associated with different aspects of their daily life, but also the unique challenges faced by those living in rural areas. The stigma surrounding having a child with CCNs was reported by 2 participants and was seen as a contributing factor to their burden. These mothers felt judged by others:

We wouldn’t bring Oliver in stores because he would have meltdowns and we would have to explain ourselves, and when you’re not able to control your child in the stores, well, you’re judged (. . .) (Anna).

The caregiver burden sometimes led to anxiety, exhaustion, and relationship issues: “like, as a parent my nerves in the past two years (. . .). I am on medication now for it, but it is a lot on the head I guess” (Melissa). To that she added:

I am always on edge, like my anxiety, especially if we leave the house, like, even when we go to speech therapy or doctor’s appointment, in the waiting room, I don’t know if he’s going to hit the kids (. . .) I am on edge, really, all the time, every day (. . .).

Having a child with CCNs requires mothers to play multiple roles to meet the child’s needs: “I don’t play the mom role very often, so I wish I could have more like better times with Jack than always being the teacher, being the caregiver, the punisher” (Melissa). For some mothers, like Charlotte, the caregiver burden seemed shared with her husband, but the high level of engagement from both parents left no time to invest in their couple: “Well, probably it wouldn’t be a bad thing if Jacques and I could have more time to ourselves, because I think we do too much (. . .) it’s more for the children and we’re not working on our relationship.” Finally, one mother explained that working part-time could help alleviate her caregiver burden, but for financial reasons, it is not possible.

Another important challenge contributing to caregivers’ burden in rural areas is the difficulty of accessing resources and services. For instance, Anna explains: “(. . .) often when you have challenges with kids like Oliver you often have to fight to find the resources, to find help.” In rural areas, even when services are available, the shortage of qualified professionals can make them inaccessible. For instance, Charlotte explained: “Well, there was respite (. . .) and sometimes you have to look for that help, but how do you find someone who would be willing and who would be good with him?”. This highlights the difficulties rural families face in securing reliable and high-quality support, even when services technically exist. Valery expressed that the limited human resources in school made it hard for the teacher to implement the student’s personalized learning plan: “I find it hard (. . .) I think there’s a lack of resources and I really don’t think that the intervention plan can be respected like it should be.” In addition, the pandemic also brought new challenges related to accessing services. One mother reported that finding sporting activities for her son was harder due to restrictions and closures. These examples underscore the challenges rural mothers face in accessing adequate support for their family.

## Discussion

This study aimed to understand the experiences and challenges of mothers caring for a child with CCNs and living in rural settings. The rural context presents unique barriers, including limited access to healthcare services and resources, which can exacerbate the difficulties mothers face. Our results showed multifaceted challenges related to children’s needs and their impact on the mother’s well-being. Even though mothers experienced a wide range of daily challenges related to their child’s or family functioning, they also reported similar struggles in understanding and managing their children’s behaviors and diagnoses. These findings are in line with a similar study conducted in urban settings, where mothers’ reactions to their child’s diagnosis and the lack of knowledge on how to manage their child’s health issues were contributing factors to their emotional burden.^7^

Although our study did not have a quantitative component to measure specific indicators related to the children’s diagnosis and associated challenges, the qualitative data showed that all children encountered major challenges, with some requiring significant involvement from their mothers. The intensity of children’s symptoms was a primary stressor for caregivers, with more severe symptoms often leading to an increased perceived burden. Our results also show that managing the caregiver burden on a daily basis was particularly difficult for many mothers. Additionally, mothers reported difficulties in balancing multiple roles and responsibilities, along with the added burden of facing judgments from others, all of which contributed to the heightened caregiver burden. This aligns with previous qualitative studies^3,30^ conducted with urban, suburban, and rural families, which highlighted that parents having children with disabilities face challenges (ie, caregiving responsibilities, access to resources, financial cost) that can lead to considerable personal distress and can negatively impact both the child and the family well-being. In our study, the compounded effect of living in a rural setting (transportation barriers and fewer community resources^15^) and having a child with CCNs seemed to have further intensified their caregiver burden.

Another important factor to consider when working with mothers and children living with CCNs in rural settings is the quality of the support network. Studies have shown that both formal and informal support systems play a crucial role to diminish caregiver burden of parents and families.^31,32^ The results from our study indicate that mothers felt inadequately supported in accessing formal resources, such as support services, specialized healthcare, and mental health services offered through centralized public services, often located in urban settings. This may be attributed to a lack of information about services available outside the CSP center, as reported by the mothers. These findings are consistent with prior studies highlighting that parents of children with CCNs often report insufficient knowledge and limited information about available resources and how to access them.^3,33,34^ In rural settings, these challenges are often intensified by limited access to health and psychosocial services, making it even more difficult for mothers to access the necessary support to care for their child with CCNs.^35^ Moreover, families often encounter a complex system of care, characterized by long wait lists, fragmented services, and language barriers, all of which have been shown to complicate the navigation of the care system.^36^

Overall, results suggest that the ICIM^22^ might be useful to consider which factors might be influencing caregiver burden when working with mothers having a child with CCNs and living in rural settings. When applying this framework to our current results, we can suggest that health determinants (ie, access to formal and informal support, perceived stigma), stressors (ie, intensity of symptoms, multiple roles), and individual characteristics (ie, knowledge about the children’s challenges) were expressed by mothers and shown to impact their perceived caregiver burden. By integrating the ICIM^22^ into practice, healthcare providers would get a better understanding of how these factors intersect and affect mothers’ well-being and burden, which is especially relevant when working with mothers having limited access to support. Furthermore, this approach could be helpful to develop more tailored and context-sensitive interventions for mothers living in rural settings, with the goal of reducing their caregiver burden.

### Clinical Implications

To reduce the burden of mothers of a child with CCNs, pediatric interventions should be tailored and centered on the child and the family’s specific needs, particularly in rural areas where access to specialized care can be limited.^15^ Some research has shown the positive impact of parent interventions (eg, parent-child interaction therapy, didactic and pyramidal training) on children’s outcomes (eg, disruptive behavior, social awareness)^37,38^ and the possibility to remotely deliver these interventions to families living in rural communities.^39^ However, less is known about the impact of these interventions on parents’ outcomes.^40^ A recent meta-analysis examining the impact of parent interventions reported that parents of children with autism spectrum disorder benefited primarily with regard to their parenting confidence and mental health, but no significant improvement was observed with their caregiver burden.^40^

In rural areas, where healthcare services are often less accessible and there are fewer specialized resources,^15,35^ offering interventions tailored to mothers’ needs could be especially important to reduce their burden. One universal intervention is unlikely to meet the needs of every mother, particularly given the diverse challenges, cultural background, or even geographic location.^41^ Edelstein et al’s^42^ scoping review highlighted that care coordinators can play an essential role in decreasing caregiver burden in providing administrative, psychosocial, and emotional support. Concretely, there are educational programs that target healthcare providers and their ability to communicate, evaluate caregivers’ needs, and help them navigate the resources.^43^ Currently, in many rural contexts, the responsibility of care coordination often falls to parents or guardians,^44^ thus increasing their burden. For mothers of children with CCNs, a care coordinator could be instrumental in helping them navigate services that might be scattered over large distances (eg, respite care, peer support), and offering intervention tailored to their specific needs.^42,45^ This could improve their ability to support families with limited access to services and help reduce their burden. Considering the current results showing mothers’ challenges in accessing reliable and high-quality services for their children, we believe that in addition to increasing available services in rural settings, having access to care navigation services could support mothers’ advocacy and search efforts and lead to an easier and more sustainable access to care.

### Limitations of the Study

This study has some limitations. The small sample size (N = 8) limits transferability of findings, and the sampling strategy, which focused on families who were receiving services from the CSP Centre, is not representative of all mothers with children having CCNs in rural settings. Additionally, while there were no exclusion criteria for fathers or significant others, our recruitment strategy unintentionally resulted in the inclusion of only mothers. We recognize that a more tailored recruitment approach for fathers or significant others could have ensured more equitable participation. Finally, the demographic questionnaire and the interview guide used in our study were developed specifically for the purposes of this research. Since we were gathering information regarding multiple domains certain aspects, such as the type of informal and formal support accessed, could not be fully explored. It is nevertheless one of the only known qualitative study focusing on this population. It is also important to note that the interviews were conducted during the COVID-19 pandemic, which could have influenced the results, since the sanitary restrictions had a negative impact on mothers’ burden and social support^46^ as well as on service delivery.^47^ Future research studies should be conducted to replicate these results and continue better understanding the psychological and emotional impacts of mothers, fathers, and significant others caring for a child with CCNs in rural settings, and how to improve the support they need.

## Conclusion

The present study aimed at exploring the experiences and challenges faced by mothers of a child with CCNs living in rural communities. Findings highlighted that children’s challenges are sometimes difficult to manage and can negatively influence mothers’ burden. Access to formal resources was also difficult for mothers participating in our study. Increasing formal and informal supports available in rural settings is recommended to better meet the needs of mothers and families with CCNs.

## Supplemental Material

sj-docx-1-gph-10.1177_30502225251332024 – Supplemental material for Mothers’ Experiences Raising Children With Complex Care Needs in Rural Settings: A Qualitative Study Supplemental material, sj-docx-1-gph-10.1177_30502225251332024 for Mothers’ Experiences Raising Children With Complex Care Needs in Rural Settings: A Qualitative Study by Hélène Corriveau, Penelopia Iancu, Anik Dubé and Vickie Plourde in Sage Open Pediatrics
