# Supplementary material for: Mothers’ Experiences Raising Children With Complex Care Needs in Rural Settings: A Qualitative Study
Source: Sage Open Pediatr. 2025 May 2;12:30502225251332024. doi: 10.1177/30502225251332024 (PMC12220894; doi:10.1177/30502225251332024)
Supplement: sj-docx-1-gph-10.1177_30502225251332024 – Supplemental material for Mothers’ Experiences Raising Children With Complex Care Needs in Rural Settings: A Qualitative Study [file sj-docx-1-gph-10.1177_30502225251332024.docx]

**Section reserved for the research team**

Identification no. family :­­­­__________

Date completed (day/month/year) :__________

**
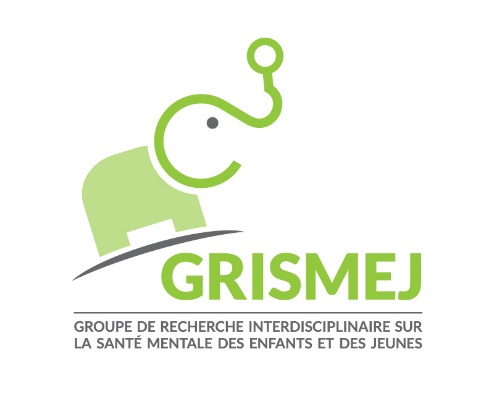
**

**Questionnaire for parents, community social pediatrics**

**Confidentiality reminder:** As a reminder, your participation in the study will remain confidential, meaning that no one, other than the members of the research team, will have access to personal information.

**Context setting**

- Verify: Number of children followed, date of beginning of services, accompanied by which member of the social pediatrics team

**Changes observed with their child (individual and relational dimension)**

1. What brough you to seek help? Were you referred by another service?

2. Tell me about the changes you have observed in your child since you have accessed this program (community social pediatrics).

3. Tell me about how he/she feels (emotions, self-confidence, etc.). How do you find your child's behavior since you have been using the community social pediatric services? Tell me about that.

4. How do you get along with your child? Tell me about the changes you have observed since he receives community social pediatric services.

5. How would you describe your child's relationships with his or her siblings (if applicable)? With friends in and out of school.

**Living conditions**

1. What do you find difficult about your child's health?

2. What would help him or her to deal with the problems?

3. What would you change in your living conditions to improve your well-being?

**Children’s Rights**

Introduce this theme

1. What comes to your mind when I say “the rights of the child” (what does it mean to you)?

2. What would you like to know (or learn) about children's rights?

**Engagement with children’s education**

1. How would you describe your possibility to be involved in your child's education?

**Satisfaction with services**

1. How would you describe your experience with the services received for your child (community social pediatrics)?

2. What challenges did you encounter with the services or what things did you like less?

3. What did you enjoy most about this experience?

4. How did the services received help you in your daily life?

5. What did you find different compared to other services?

6. What would you like to change regarding these services (suggestions to keep improving the services)?
